# Catechin-capped silver-doped titanium dioxide nanoparticle enhanced photocatalytic toxic dye degradation

**DOI:** 10.3389/fchem.2025.1576504

**Published:** 2025-04-15

**Authors:** Sougata Ghosh, Tanawat Imboon, Rashbihari Layek, Gayatri Salunke, Vijay Singh Parihar, Jeerawan Khumphon, Thomas J. Webster, Santosh Sutar, Sutasinne Kityakarn, Chaisak Issro, Dusadee Khamboonrueang, Sirikanjana Thongmee

**Affiliations:** ^1^ Department of Physics, Faculty of Science, Kasetsart University, Bangkok, Thailand; ^2^ Department of Microbiology, School of Science, RK. University, Rajkot, Gujarat, India; ^3^ Department of Physics, National Institute of Technology Durgapur, Durgapur, West Bengal, India; ^4^ Biochemical Sciences Division, CSIR-National Chemical Laboratory, Pune, Maharashtra, India; ^5^ Academy of Scientific and Innovative Research (AcSIR), Ghaziabad, Uttar Pradesh, India; ^6^ Biomaterials and Tissue Engineering Group, Faculty of Medicine and Health Technology, Tampere University, Tampere, Finland; ^7^ School of Health Sciences and Biomedical Engineering, Hebei University of Technology, Tianjin, China; ^8^ School of Engineering, Saveetha University, Chennai, India; ^9^ Materials Program, Federal University of Piaui, Teresina, Brazil; ^10^ Yashwantrao Chavan School of Rural Development, Shivaji University, Kolhapur, Maharashtra, India; ^11^ Department of Chemistry, Faculty of Science, Kasetsart University, Bangkok, Thailand; ^12^ Department of Physics, Faculty of Science, Burapha University, Chonburi, Thailand; ^13^ Faculty of Science and Technology, Nakhon Sawan Rajabhat University, Nakhon Sawan, Thailand

**Keywords:** green synthesis, catechin, titanium dioxide nanoparticles, silver doping, photocatalysis, methylene blue dye, rhodamine B

## Abstract

Doping-associated surface modification is a powerful strategy to enhance the photocatalytic potential of n-type semiconductor nanomaterials. Silver (Ag) is one of the most effective dopants that can result in the retardation of the electron hole recombination-generating Schottky barrier at the TiO_2_ interface with a simultaneous extension of absorption to the visible region. This work presents a study on the effect of catechin, a bioactive principle polyphenol compound found in various plants, on the synthesis, Ag-doping and stabilization of TiO_2_ nanoparticles (TiO_2_NPs). The nanoparticles were irregular in shape with sizes ranging from 19 to 30 nm. Ag-TiO_2_NPs were fabricated using TiO_2_ as a precursor and 1%, 3%, and 5% AgNO_3_ as a doping agent. The average particle size of 1%Ag-TiO_2_NPs, 3%Ag-TiO_2_NPs, and 5%Ag-TiO_2_NPs was 27.3 ± 7.5 nm, 29.8 ± 9.6 nm, and 25.0 ± 9.0 nm, respectively. High-resolution transmission electron microscopy (HRTEM) showed lattice fringes with an interplanar spacing of 0.23 nm corresponding to the Ag (111) plane in addition to the presence of the anatase phase of TiO_2_. Fourier transform infrared (FTIR) spectra exhibited a broad peak around 400–800 cm^−1^ that was attributed to Ti-O-Ti stretching vibrations which was slightly shifted in Ag-TiO_2_NPs due to changes in the local bonding environment around Ti atoms caused by interactions with Ag. Catechin loading in the TiO_2_NPs and Ag-TiO_2_NPs was between 1.55 and 3.3 wt. %. TiO_2_NPs, 1%Ag-TiO_2_NPs, 3%Ag-TiO_2_NPs, and 5%Ag-TiO_2_NPs exhibited superior photocatalytic degradation of methylene blue dye up to 78%, 87%, 91%, and 92%, respectively, and RhB dye up to 92%, 94%, 97% and 99%, respectively, with a pseudo-first-order reaction kinetics. Furthermore, its recyclability was also demonstrated for three cycles. The simplicity of fabrication and superior photocatalytic performance of TiO_2_ demonstrated here make this green route advantageous for environmental applications to treat dye contaminated effluent as well as for numerous other applications.

## Graphical Abstract

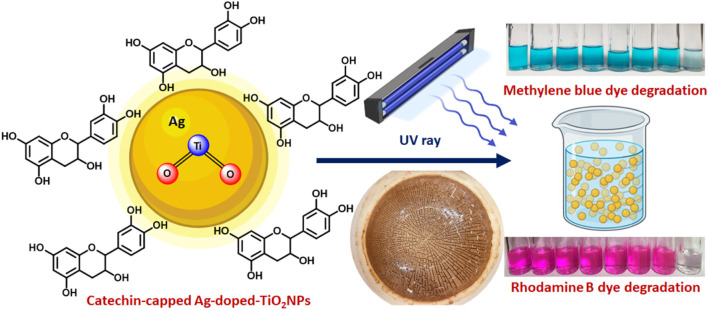

## 1 Introduction

Unlike pigmentary titanium dioxide (TiO_2_), nanoscale TiO_2_ is smaller in size (<250 nm) and exclusively used in photocatalysis and/or in UV filters. Their broad spectrum applications as additives in cosmetics, electrochromic and electronic devices, food, paints, plastics, photo voltaic cells, sensors, and sunscreens have resulted in its large production which is around 10,000 tons globally each year (Ayorinde and Sayes, 2023; Kader et al., 2022; Liza et al., 2024). Conventionally, titanium dioxide nanoparticles (TiO_2_NPs) are synthesized using microwave-assisted deposition with electrophoresis, thermal plasma and spray pyrolysis, chemical co-precipitation, microemulsion hydrothermal, solvothermal, and sol-gel methods (Wang et al., 2022; Sagadevan et al., 2022; Jassal et al., 2022; Arun et al., 2023; Irshad et al., 2021; Mahdi et al., 2023; Narh et al., 2024). However, these methods are often expensive and involve hazardous toxic chemicals for synthesis and stabilization. Hence, the biocompatibility of TiO_2_NPs is compromised. When using such toxic agents, they can exhibit toxicity against various organs such as the gastrointestinal tract, kidney, liver, lungs, skin, and spleen (Chen et al., 2009). Thus, there is a need to develop a green route for the economical synthesis of TiO_2_NPs.

More recently, biological extracts of bacteria, fungi, algae, and plants have been used as reducing agents for synthesizing TiO_2_NPs (Pandya et al., 2024; Rathi and Jeice, 2024; Rathi et al., 2023). Although it is advantageous to physically and chemically synthesize green NPs using such biological extracts, the complex mixture of metabolites in the extracts results in an unpredictable size and shape of the NPs. Thus, pure bioactive principles may serve as a potential solution for the synthesis of stable and uniform TiO_2_NPs.

Plant extracts are a rich source of a diverse group of flavonoids and polyphenols that include caffeic acid, catechins, epicatechin gallate, epicatechin, epigallocatechin gallate, epigallocatechin, gallaocatechin, gallate, gallic acid, quercetin, rutin, and salicin (Golpour et al., 2024; Vaishnavi et al., 2023; Alavi et al., 2023). Catechin is the most predominant bioactive principle in several medicinal plants that has profound therapeutic benefits. Preliminary studies suggest that catechin can be used to synthesize spherical gold nanoparticles (AuNPs) 53 nm in size (Ebrahimian et al., 2022). Likewise, a green tea extract, thought to be rich in catechin, was used for the synthesis of silver nanoparticles (AgNPs) 91 nm in size which showed anticancer activity against melanoma cells (Golpour et al., 2024). However, despite promise, there are no reports on the catechin-mediated synthesis of TiO_2_NPs.

The metal doping-mediated enhancement of photocatalytic properties of semiconductors is a promising strategy (Khodabandeloo et al., 2023; Liu and Chu, 2023; Ridha et al., 2021). For example, TiO_2_NPs are often rationally doped with metals and/or non-metals to improve their photoactivity (Zhao et al., 2021; Shan et al., 2023). Thus, doping with Ag may serve as electron traps, thereby enhancing electron-hole separation (Singh and Kumar, 2021; Skiba et al., 2021; Aravind et al., 2022; Periyasamy et al., 2024). It can result in the extension of light absorption into the visible range which in turn can promote the excitation of surface-associated electrons due to visible light-mediated plasmon resonance excitation. This strategy may even alter the surface properties of the TiO_2_NPs and result in synergistic enhancement of photocatalytic potential (Abbad et al., 2020).

In view of this background, we report here for the first time, the synthesis of catechin-functionalized TiO_2_NPs and Ag-doped TiO_2_NPs which were characterized using various analytical techniques. Further, their photocatalytic dye degradation properties were checked and confirmed.

## 2 Materials and methods

### 2.1 Chemicals and reagents

Catechin hydrate (HPLC grade) was procured from Sigma-Aldrich, USA. Titanium dioxide (TiO_2_) was purchased from Sisco Research Laboratories (SRL) Pvt. Ltd., India while silver nitrate (AgNO_3_), methylene blue, and rhodamine B dye were purchased from HiMedia Laboratories Pvt. Ltd., India. Ethylenediaminetetraacetic acid disodium salt (EDTA-Na_2_) was procured from Ajax Finechem Pty Ltd, Australia.

### 2.2 Synthesis of TiO_2_NPs and Ag-TiO_2_NPs

50 mL of a reaction mixture containing a 5 mM catechin and 0.5 M TiO_2_ salt solution was prepared and subjected to stirring for 24 h at room temperature for the synthesis of TiO_2_NPs. The change in the color of the reaction mixture from white to pink-brown indicated the production of TiO_2_NPs. The reaction mixture was centrifuged at 5,000 rpm for 10 min for the separation of TiO_2_NPs. The resultant pellet was washed and dried at 60°C for 24 h in a hot air oven. Ag-TiO_2_NPs were synthesized by taking varying concentrations (1%, 3%, and 5%) of Ag from the composites. In short, the reaction mixtures containing 5 mM of catechin and 0.5 M TiO_2_ salt solutions were supplemented with 5 mM, 15 mM, and 25 mM AgNO_3_ to obtain 1%, 3%, and 5% Ag doping, respectively. The solution was stirred at room temperature for 24 h followed by washing by centrifugation at 5,000 rpm for 10 min and redispersion in deionized (DI) water. The resulting NPs were dried as mentioned earlier.

### 2.3 UV–vis spectroscopy

The synthesis of the NPs was confirmed using UV–vis spectroscopy (Shimadzu 1800 UV–vis spectrophotometer, Japan). An aqueous suspension of catechin-synthesized TiO_2_NPs and Ag-TiO_2_NPs was prepared at a concentration of 1 mg/mL and the UV–vis spectra were recorded at a range between 200 and 800 nm. The optical energy band gaps (E_g_) of the NPs were calculated from the (αh*ν*) (Alavi et al., 2023) versus photon energy (h*ν*) Tauc plots.

### 2.4 Morphological and elemental characterization

The shape and size of the NPs were investigated using a FEI Quanta 450 scanning electron microscope (SEM). Elemental mapping for Ti, O, and Ag was carried along with energy-dispersive X-ray spectroscopy (Oxford Instruments X-max 50 mm^2^) to confirm the elemental composition. The NPs were loaded onto carbon-coated copper grids and subjected to high-resolution transmission electron microscopy (HRTEM) using a JEOL JEM 3100F field emission transmission electron microscope (FE-TEM) that operated at 300 kV (Ridha et al., 2021).

### 2.5 Particle size and zeta potential analysis

Aqueous solutions of NPs (1 mg/mL) were sonicated for 30 min before measuring their hydrodynamic size. The mean particle size, polydispersity index (PDI), and zeta potential of the TiO_2_NPs and Ag-TiO_2_NPs were determined by a dynamic light scattering (DLS) method using a 90 Plus nanoparticle size and zeta potential analyzer (Brookhaven Instruments Corp., USA).

### 2.6 X-ray diffraction and fourier-transform infrared spectroscopy

XRD pattern analysis using an AXS D8 Focus P-XRD, Bruker operating at a voltage of 40.0 kV and a current of 40.0 mA was used to assess the crystallinity of the undoped and the Ag-doped TiO_2_NPs. The FTIR spectra were recorded in a range of 400–4,000 cm^−1^ using a PerkinElmer FTIR spectrophotometer, UK (Bloch et al., 2022).

### 2.7 Thermogravimetric analysis

About 2 mg of pure catechin hydrate and NPs were added to the sample plate and heated at a rate of 20°C/min. The temperature range was 25°C–600°C. The reduction in mass was determined by a PerkinElmer STA 6000 thermogravimetric analyzer. The thermogravimetric analysis (TGA) curve was drawn and the results were analyzed.

### 2.8 Photocatalytic dye degradation

The dye removal efficiency of our prepared samples was investigated by exposing the MB dye solution to UV light (24 W). This experiment was done by dissolving 20 mg of each NP into 100 mL of the 10 mg L^–1 ^MB solution. After that, the resultant solution was stirred using a magnetic stirrer for 1 h in darkness at room temperature to achieve the adsorption/desorption equilibrium condition. Then, 5 mL of solution was collected and the NPs were separated by centrifugation at 8,000 rpm for 5 min. The photocatalytic experiment was performed by irradiating the UV-A light source under vigorous stirring followed by the collection of samples at regular intervals from 1 to 24 h. After separating the NPs by centrifugation at 8,000 rpm for 5 min, the absorbance was recorded using a Shimadzu 1,800 UV–vis spectrophotometer to evaluate the dye degradation efficiencies (Nandasana et al., 2024). The impact of 1 mM EDTA-Na_2_, as a scavenger, on the photocatalytic performance of the NPs with the maximum photocatalytic activity was studied (Verma et al., 2019).

The recyclability of NPs exhibiting the highest photocatalytic activity was evaluated through three successive photodegradation cycles. After completion of each cycle, the NPs were recovered by centrifugation and washed three times before they were used for the next cycle of dye degradation. Percentage MB and RhB dye degradation was evaluated at the end of each cycle. The stability was checked by recording the UV-visible spectra of the NPs recovered at the end of the third cycle (Palanisamy et al., 2020; Chen et al., 2024).

### 2.9 Statistical analysis

To see the impact of samples (s = 4) and time points (t = 7) on the degradation process, two-way analysis of variance (ANOVA) was conducted. The result of the two-way ANOVA for photocatalytic methylene blue dye degradation was considered statistically significant when the p-value was less than 0.05 (*p < 0.05).

## 3 Results and discussion

### 3.1 UV–visible spectroscopy

**FIGURE 1:**
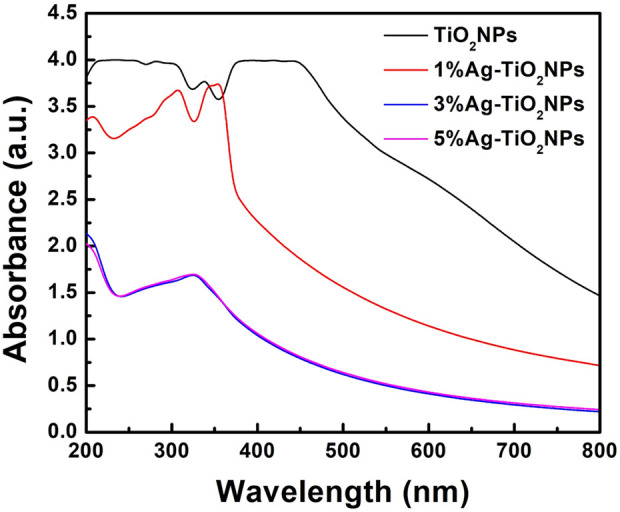
The UV–vis absorbance spectra of TiO_2_NPs and Ag-TiO_2_NPs.

The UV–vis absorbance spectra of TiO_2_NPs and Ag-TiO_2_NPs recorded in a range between 200 and 800 nm are compared in Figure 1. TiO_2_NPs exhibited an absorption peak within the 350–500 nm range, while the 1%Ag-TiO_2_NPs showed absorption peaks predominantly in the UV region, spanning 250–400 nm. In contrast, both 3%Ag-TiO_2_NPs and 5%Ag-TiO_2_NPs demonstrated absorption in the UV region, with the highest absorption observed at 329 nm. Our results are well in agreement with earlier reports where TiO_2_NPs synthesized using *Echinacea purpurea* herbal extract showed UV-vis peaks between 200 and 400 nm (Dobrucka, 2017). Similarly, another study showed the absorbance maxima of TiO_2_NPs prepared using *Euphorbia heteradena* Jaub root extract was 360 nm (Nasrollahzadeh and Sajadi, 2015).

Surface plasmon resonance (SPR) plays a crucial role in determining optical properties and associated applications. The SPR effect highly depends on particle size, shape, and the surrounding dielectric environment. Similar alteration of the SPR was also noted when ZnS QDs were incorporated in the ZnS/Ag/CoFe_2_O_4_ nanocomposite where the absorbance in the UV region was enhanced. Thus, the composite exhibited superior absorption in both visible and UV light. This enhanced optical absorption may result in better charge carrier separation, which is evident from the photocatalytic dye degradation included in the application part (Palanisamy et al., 2020). An increase in the absorbance intensity also depends on the thickness of the silver film which also involves a blue shift in the absorption peak. This was noted when AgNPs were incorporated in TiO_2_NPs containing dye-sensitized solar cells (DSSCs) where it was speculated that the alteration of the SPR was dependent on the size of the AgNPs (Li et al., 2023).

**FIGURE 2:**
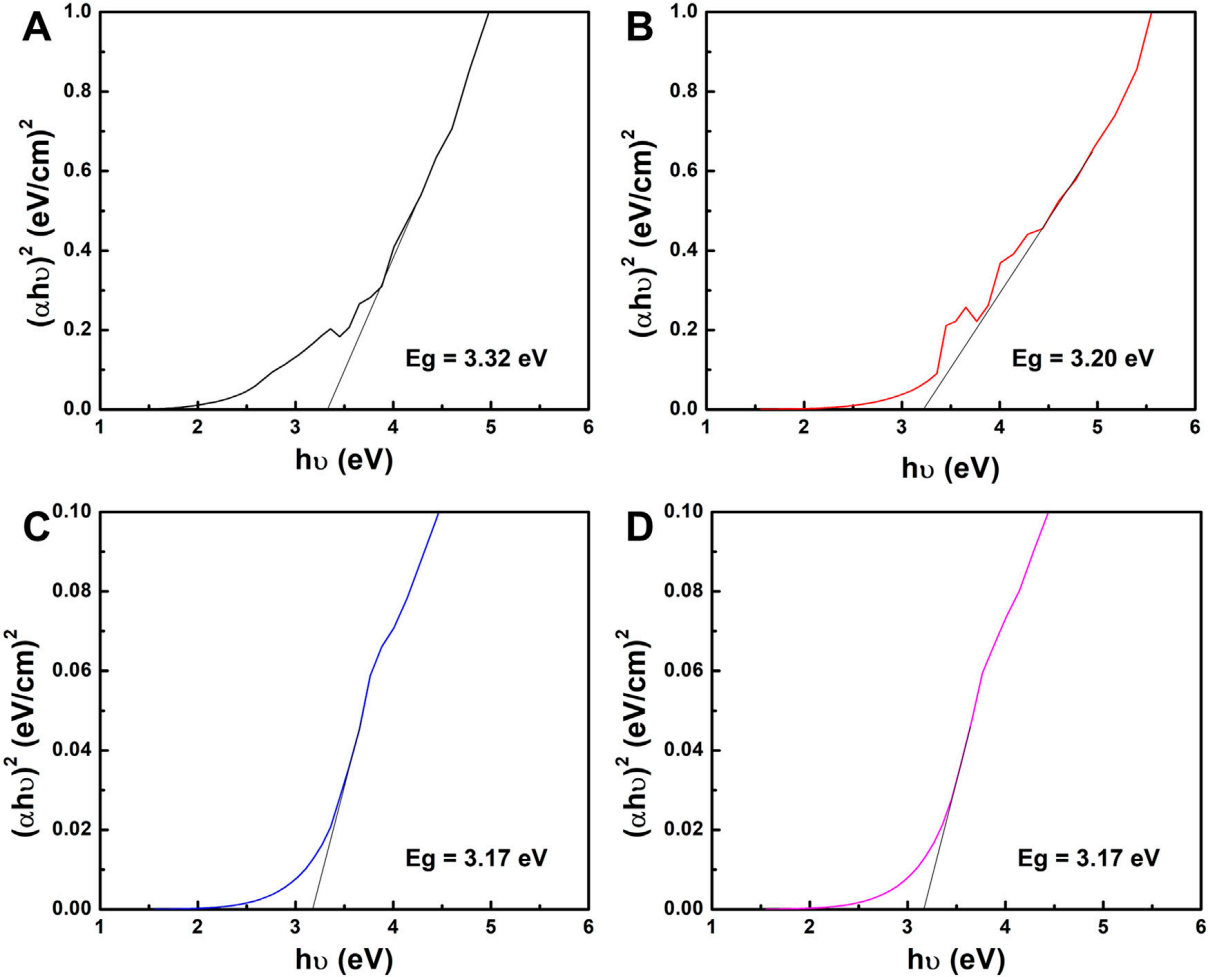
Tauc plot of **(A)** TiO_2_NPs; **(B)** 1%Ag-TiO_2_NPs; **(C)** 3%Ag-TiO_2_NPs; and **(D)** 5%Ag-TiO_2_NPs.

Furthermore, the UV–vis absorbance spectra were used to estimate the energy band gap of the samples as shown in Figure 2. The energy band gap was determined using the Tauc plot and were 3.32, 3.20, 3.17, and 3.17 eV for TiO_2_NPs, 1%Ag-TiO_2_NPs, 3%Ag-TiO_2_NPs, and 5%Ag-TiO_2_NPs, respectively. Notably, the results indicated a slight decrease in the energy band gap for the Ag-doped samples compared to pure TiO_2_NPs. This observation aligns with the previous report where the band gap of neat TiO_2_ (3.36 eV) reduced to 3.07 eV and 2.5 eV after Ag and CuO doping (Hazarika et al., 2023). The reduction in the energy band gap with increasing Ag content suggests that the presence of Ag species may inhibit the recombination of charge carriers generated under UV light irradiation within the TiO_2_ crystal lattice, thereby enhancing the material’s catalytic activity. Nyankson et al. (2022) reported a similar reduction in the energy band gap for curcumin-loaded Ag–TiO_2_-halloysite nanotubes (Nyankson et al., 2022).

### 3.2 FE-SEM, EDS, and elemental mapping

**FIGURE 3:**
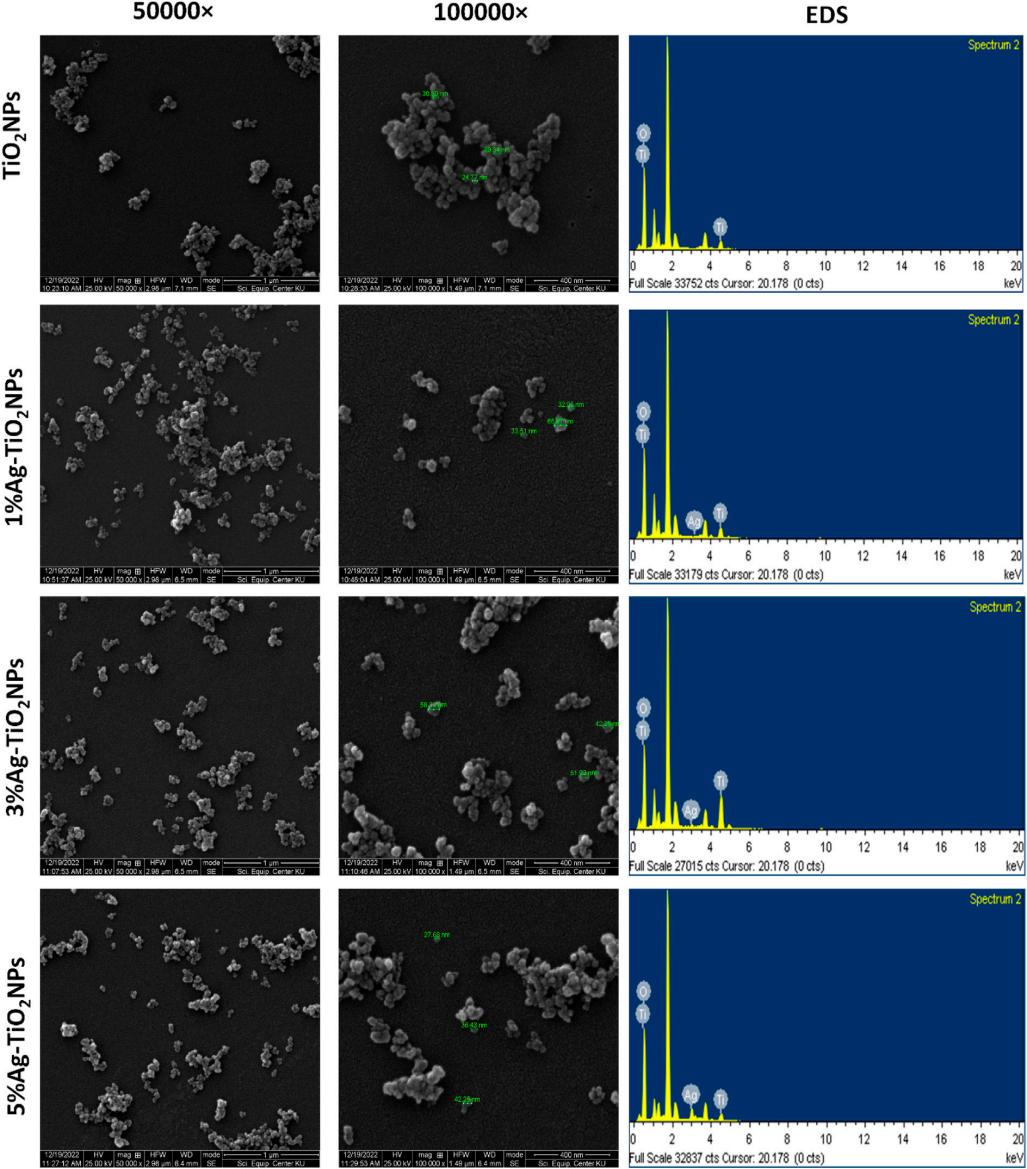
FE-SEM images at different magnifications and EDS results of the undoped and Ag-doped TiO_2_NPs.

**FIGURE 4:**
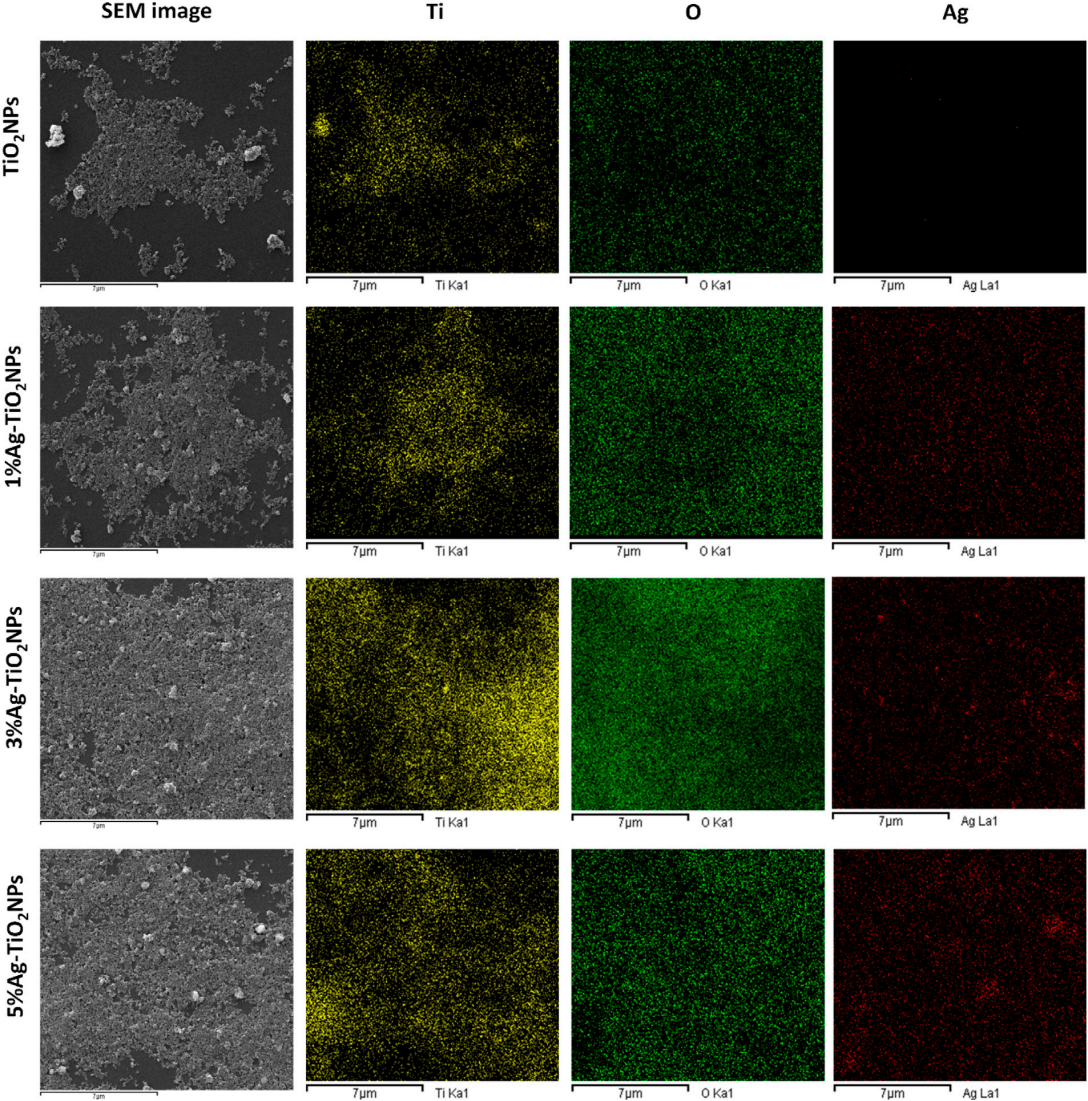
Elemental mapping of Ti (yellow), O (green), and Ag (red) in the undoped and Ag-doped TiO_2_NPs.

The shape and size of the NPs were analyzed using FE-SEM where discrete aggregates composed of a few NPs in each cluster were noted. Figure 3 shows irregular TiO_2_NPs of sizes ranging from 24.77 nm to 39.34 nm. The 1%Ag-TiO_2_NPs were 32.06 nm–65.57 nm in size while the 3% Ag-TiO_2_NPs were 42.25 nm–58.30 nm. The size of the 5%Ag-TiO_2_NPs was in the range from 27.68 nm to 42.25 nm. EDS analysis showed the elemental composition of the NPs as given in Table 1. The highest Ag content was noted in 5%Ag-TiO_2_NPs followed by 3%Ag-TiO_2_NPs, and 1%Ag-TiO_2_NPs that were equivalent to 48.17%, 5.03%, and 3.08% weight percentage and 17.99%, 1.29%, and 0.78% atomic percentage, respectively. Similar polydispersed TiO_2_NPs in aggregates were reported earlier in a study where a *Annona squamosa* peel extract was used for synthesis. The size of the phytogenic and bacteriogenic TiO_2_NPs was 23 ± 2 nm and 40–60 nm as per earlier reports, respectively (Roopan et al., 2012). Our observations are well in agreement with a previous study where the size of the NPs increased with greater Ag doping from 0.75 to 3.5 atomic % of Ag in TiO_2_NPs that were synthesized by a single-step sol−gel method (Mogal et al., 2014). Figure 4 shows the uniform dispersion of Ag in the Ag-TiO_2_NPs. The signal from Ag increased with higher doping concentration. A similar observation was made for hydrothermally synthesized Ni and Ag-doped colloidal TiO_2_NPs (Elbasuney et al., 2024).

**TABLE 1:** Elemental composition of the undoped and Ag-doped TiO_2_NPs.

NPs	Weight %			Atomic%		
	Ti	O	Ag	Ti	O	Ag
TiO_2_NPs	59.95	40.05	—	33.33	66.67	—
1%Ag-TiO_2_NPs	57.97	38.95	3.08	32.95	66.28	0.78
3%Ag-TiO_2_NPs	56.71	38.26	5.03	32.69	66.02	1.29
5%Ag-TiO_2_NPs	28.93	22.90	48.17	24.34	57.67	17.99

### 3.3 HRTEM analysis

**FIGURE 5:**
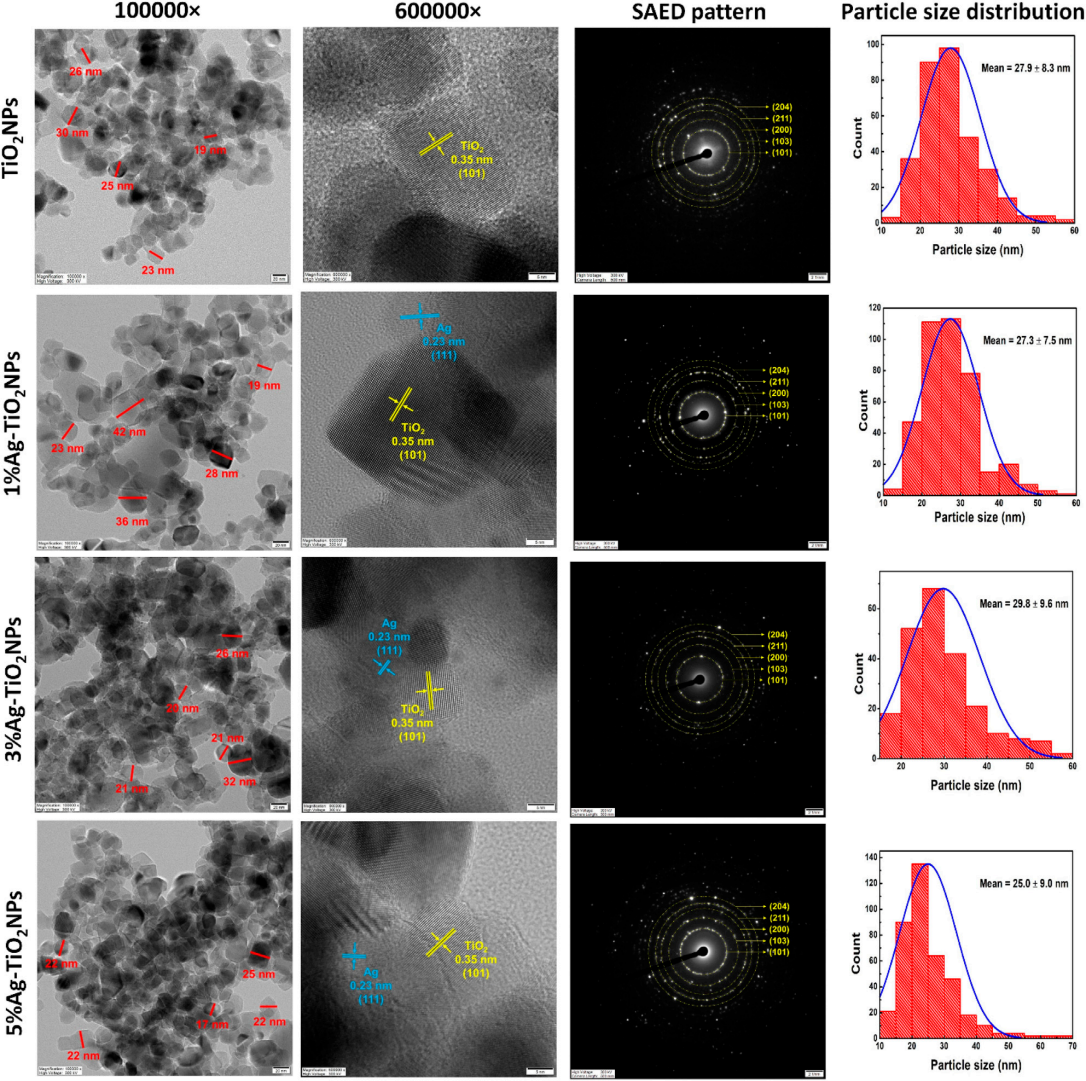
HRTEM images of monodispersed particles with dimensions, lattice fringes with d spacing, SAED pattern, and particle size distribution of undoped and Ag-doped TiO_2_NPs.

The morphology of the NPs was further analyzed using TEM as shown in Figure 5. It can be seen that the TiO_2_NPs were irregular in shape with sizes mostly between 19 and 30 nm. They were found in aggregates piling on each other. The HRTEM image showed clear lattice fringes with an interplanar spacing of 0.35 nm corresponding to the anatase TiO_2_ (101) plane. The histogram for particle size distribution showed that the TiO_2_NPs were in a range between 14.8 and 58.6 nm, with the mean particle size being 27.9 ± 8.3 nm. The larger particle size might be attributed to the agglomeration of the smaller particles. In the case of 1%Ag-TiO_2_NPs, similar irregular shapes were seen that were between 19 and 42 nm. The particle size histogram showed that the size of 1%Ag-TiO_2_NPs was between 12.8 and 59.6 nm, with the average being 27.3 ± 7.5 nm. 3%Ag-TiO_2_NPs and 5%Ag-TiO_2_NPs were also in aggregation where the size of the particles was predominantly around 20 and 30 nm. The particle size histogram showed that the 3%Ag-TiO_2_NPs were in the range between 16.5 and 56.3 nm while the average size was 29.8 ± 9.6 nm. Likewise, 5%Ag-TiO_2_NPs were between 11.0 and 69.6 nm in size while the average size was 25.0 ± 9.0 nm. HRTEM images of the Ag-TiO_2_NPs showed lattice fringes with an interplanar spacing of 0.23 nm corresponding to the Ag (111) plane in addition to anatase TiO_2_. The selective area electron diffraction (SAED) pattern further confirmed the polycrystal structure with (101), (103), (200), (211), and (204) concentric diffraction rings of anatase TiO_2_. Earlier reports with similar observations on interplanar spacing speculated the arrangement where Ag clusters of 5–10 nm were surrounded by TiO_2_NPs to form the composites (Li et al., 2017).

### 3.4 DLS and zeta analysis

**FIGURE 6:**
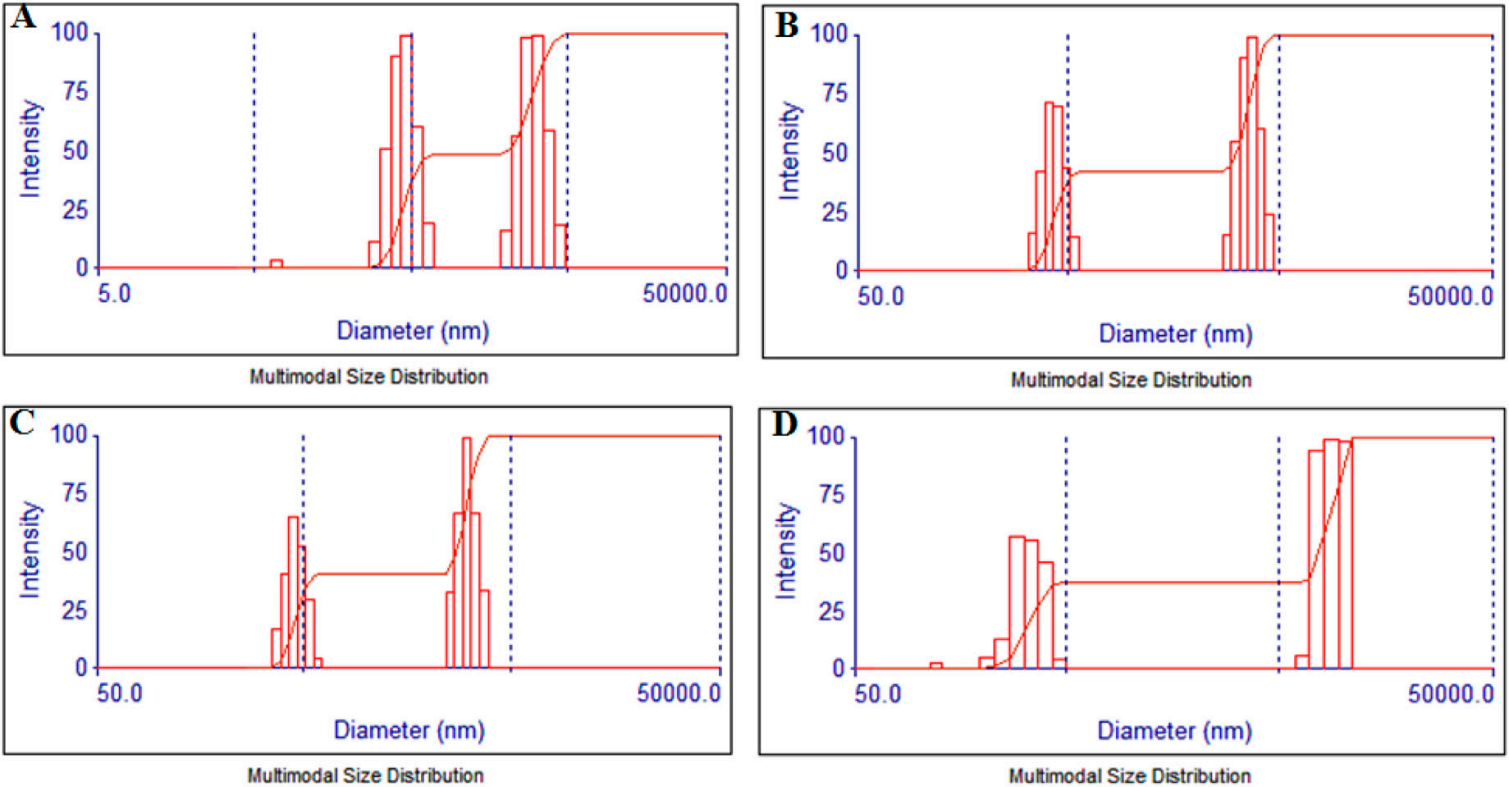
Particle size analysis by dynamic light scattering for: **(A)** TiO_2_NPs; **(B)** 1%Ag-TiO_2_NPs; **(C)** 3%Ag-TiO_2_NPs; and **(D)** 5%Ag-TiO_2_NPs.

The hydrodynamic size of the particles was evaluated employing dynamic light scattering (DLS) where the particles showed a narrow distribution in two discrete regions as shown in Figure 6. The smaller NPs showed freshly synthesized nuclei of the TiO_2_NPs that slowly aggregated together and grew in size. In the case of TiO_2_NPs, the particles were distributed in the range between 284 to 632 nm and 1,937–4,310 nm with the mean size of the aggregates being 1722.4 nm as seen in Figure 6A. Similarly, the particle size distribution for 1%Ag-TiO_2_NPs was between 333.2 nm to 527.2 nm and 2749.2 nm–4349.6 nm with the average being 2194.7 nm as evident from Figure 6B. The particle size distribution for 3%Ag-TiO_2_NPs was between 364.8 nm to 526.6 nm and 2505.5 nm–3616.4 nm, the average being 1974.1 nm as shown in Figure 6C. The particle size distribution for 5%Ag-TiO_2_NPs was between 120.7 nm and 392.3 nm and 7346.1 nm to 10 μm, the average being 5486.1 nm as seen in Figure 6D. The zeta potential for TiO_2_NPs, 1%Ag-TiO_2_NPs, 3%Ag-TiO_2_NPs, and 5%Ag-TiO_2_NPs were −14.61 mV, −10.44 mV, −15.86 mV, and −5.10 mV, respectively.

### 3.5 XRD analysis

**FIGURE 7:**
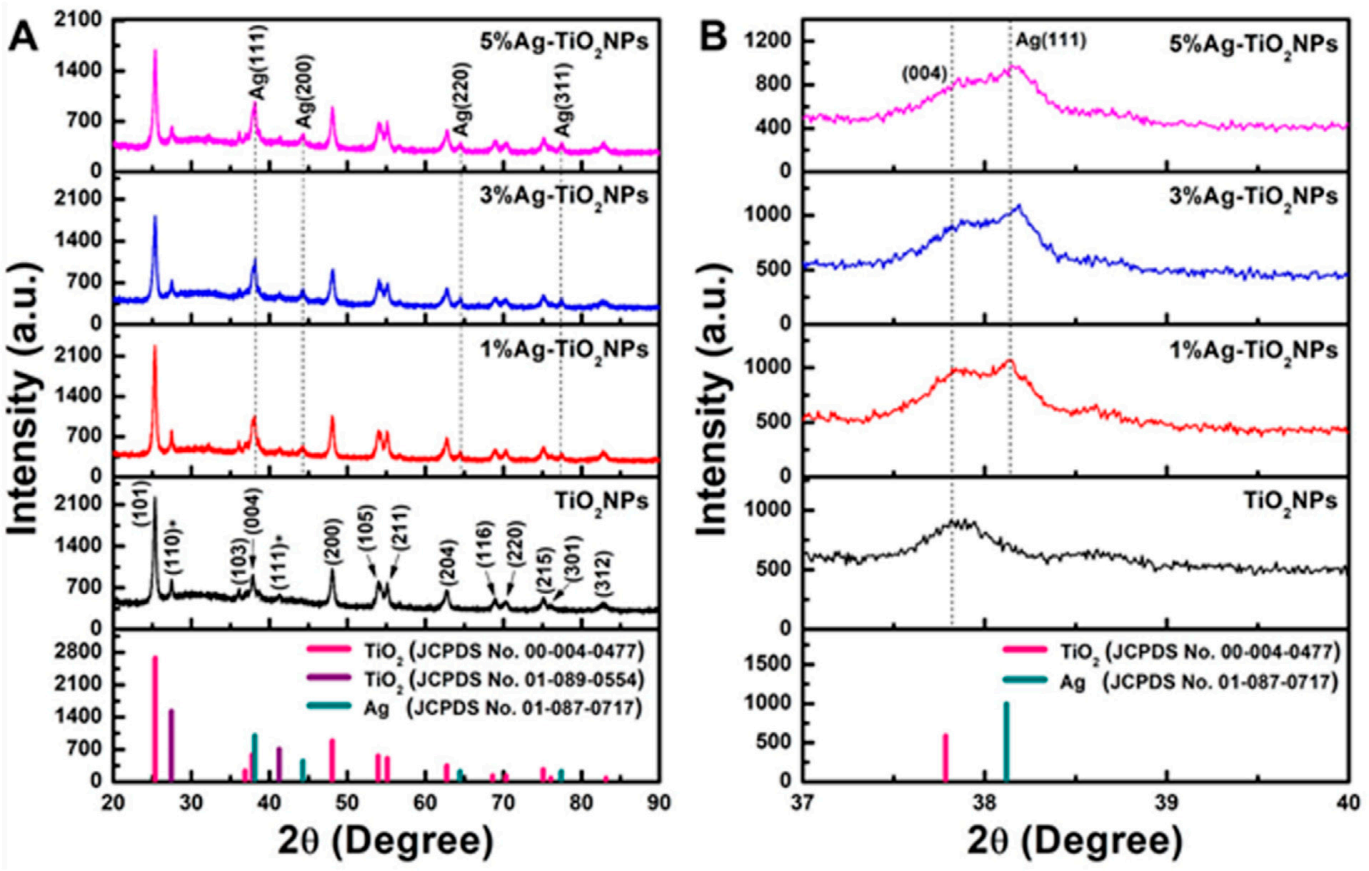
The XRD patterns of **(A)** undoped and Ag-doped TiO_2_NPs and **(B)** selected area showing the absence of Ag in pure TiO_2_NPs. Note: (*) representing peaks attributed to the rutile phase.

X-ray diffraction (XRD) analysis was conducted to estimate the crystal structure of the TiO_2_NPs and x%Ag-TiO_2_NPs (x = 1, 3, and 5 %wt.). The XRD patterns, shown in Figure 7, exhibited diffraction planes (101), (103), (004), (200), (105), (211), (204), (116), (220), (215), (301), and (312), corresponding to diffraction angles (2θ) at 25.3°, 36.1°, 37.9°, 48.3°, 54.1°, 55.1°, 62.7°, 68.9°, 70.2°, 75.1°, 75.9°, and 82.8°, respectively. These peaks confirm the anatase phase of the TiO_2_NPs, as per the standard JCPDS file No. 00-004-0477. Additionally, diffraction angles at 27.5° and 41.2° correspond to the (110) and (111) planes, indicative of the rutile phase (JCPDS file No. 01-089-0554). The XRD patterns of Ag-TiO_2_NPs revealed four additional diffraction peaks at 38.1°, 44.3°, 64.4°, and 77.4°, corresponding to the lattice planes (111), (200), (220), and (311), respectively, associated with Ag metallic particles (JCPDS file No. 01-087-0717). This confirms the presence of Ag deposition on the TiO_2_NPs surface (Khalaf et al., 2024).

Furthermore, the XRD patterns were used to estimate the crystallite size and lattice parameters of the particles (Table 2). The findings showed no significant changes in the lattice parameters. The average crystallite size was determined using the Scherrer equation, resulting in values of 15.55, 15.62, 14.56, and 14.91 nm for TiO_2_NPs, 1%Ag-TiO_2_NPs, 3%Ag-TiO_2_NPs, and 5%Ag-TiO_2_NPs, respectively. There was no significant change in the crystallite size of 1%Ag-TiO_2_NPs compared to TiO_2_NPs. However, it is noteworthy that 3%Ag-TiO_2_NPs and 5%Ag-TiO_2_NPs displayed a slight decrease in crystallite size, which may be attributed to an increase in their surface area. This reduction in crystallite size could enhance the material’s catalytic properties by providing more active sites for reactions.

**TABLE 2:** The lattice parameter and average crystallite size of undoped and Ag-doped TiO_2_NPs.

Sample	Lattice parameter (nm)			Average crystallite size (nm)
	a	c	c/a	
TiO_2_NPs	0.3783	0.9486	2.5077	15.55
1%Ag-TiO_2_NPs	0.3784	0.9486	2.5067	15.62
3%Ag-TiO_2_NPs	0.3778	0.9486	2.5107	14.56
5%Ag-TiO_2_NPs	0.3781	0.9484	2.5080	14.91

### 3.6 FT-IR analysis

**FIGURE 8:**
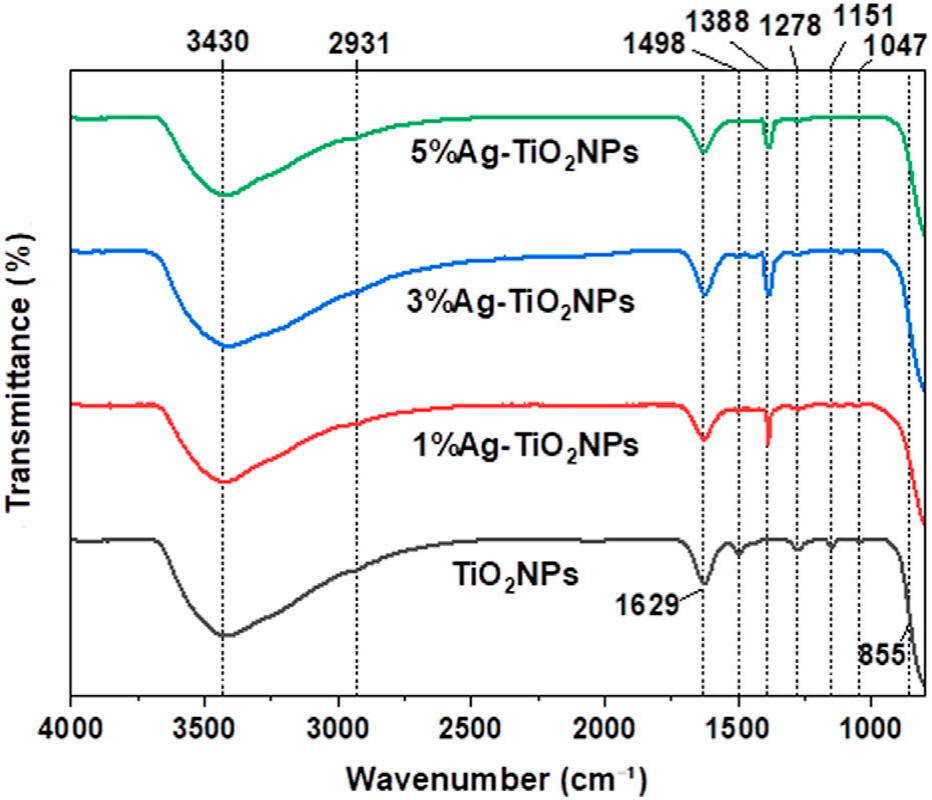
FT-IR spectra of undoped and Ag-doped TiO_2_NPs.

The FT-IR spectra of TiO_2_NPs and Ag-TiO_2_NPs in Figure 8 showed the characteristic peaks for the O-H groups (3,600–3,100 cm^−1^), C = C group stretching vibration (around 1,629 cm^−1^), C-H stretching (2,931 cm^−1^), and C–O groups (1,151–1,010 cm^−1^) which were attributed to the catechin molecules capping the TiO_2_NPs during the synthesis (Maoela et al., 2009). The broad peaks around 400–800 cm^−1^ are the characteristic of Ti-O-Ti stretching vibrations which were slightly shifted in Ag-TiO_2_NPs due to changes in the local bonding environment around Ti atoms caused by the interaction with Ag (Abbad et al., 2020). The peaks from the O-H groups merged with hydroxyl groups from residual catechin. The additional peaks at 1,388 cm^−1^ in Ag-TiO_2_NPs were due to the Ag-induced adsorption of other species such as NO_3_^−^ (nitrate groups) which usually appeared around 1,380–1,450 cm^−1^ (Mahmood et al., 2021).

### 3.7 Thermogravimetric analysis

**FIGURE 9:**
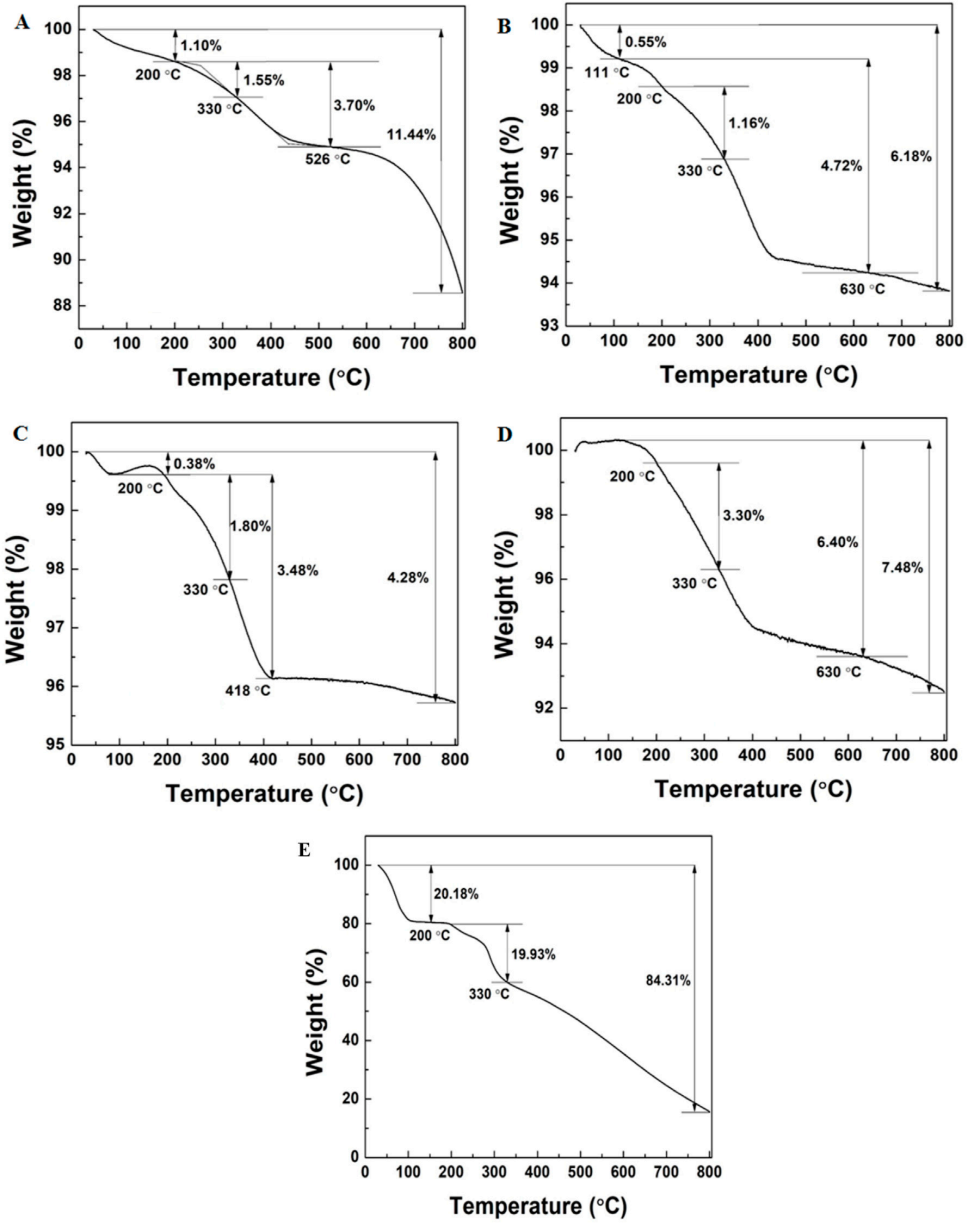
TGA analysis of: **(A)** TiO_2_NPs and **(B)** 1%Ag-TiO_2_NPs; **(C)** 3%Ag-TiO_2_NPs; **(D)** 5%Ag-TiO_2_NPs; and **(E)** pure catechin.

The TGA analysis showed the percentage loading of the catechin onto the NPs. The catechin loading on TiO_2_NPs and Ag-TiO_2_NPs with 1%, 3%, and 5% Ag doping was 1.55 wt. %, 1.16 wt. %, 1.8 wt. %, and 3.3 wt. %, respectively, as evident from Figures 9A–D. The reduction in the mass between 200°C and 330°C was due to the degradation of the catechin as seen in Figure 9E. This indicated that with the increase of the Ag doping, the association of the catechin also increased.

### 3.8 Photocatalytic dye degradation

**FIGURE 10:**
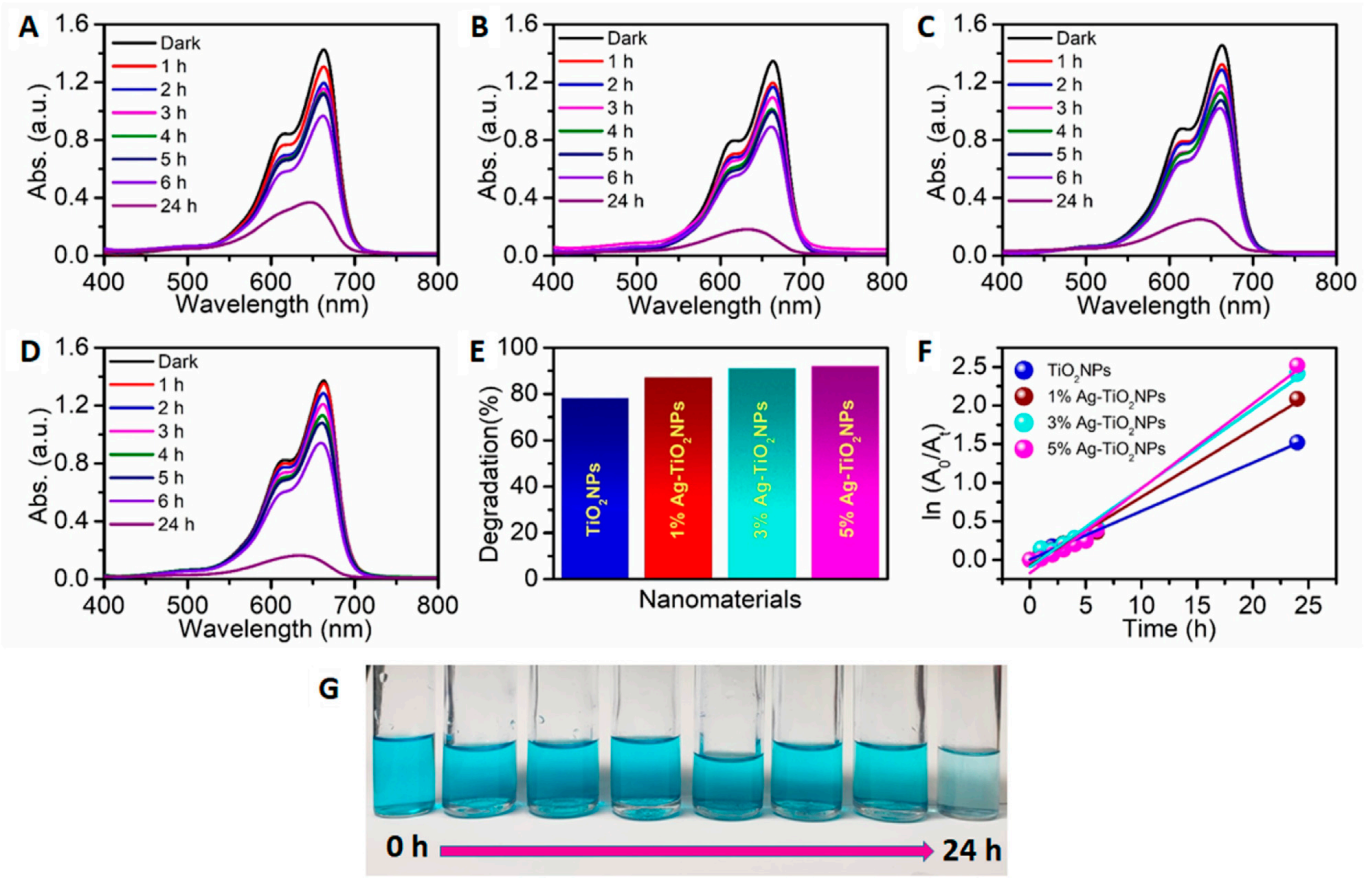
MB dye degradation under the exposure of UV light by: **(A)** TiO_2_NPs; **(B)** 1%Ag-TiO_2_NPs; **(C)** 3%Ag-TiO_2_NPs; **(D)** 5%Ag-TiO_2_NPs; **(E)** Dye removal efficiency; **(F)** Kinetic study for the photodegradation of the MB dye; and a **(G)** representative digital picture of the MB dye degradation under UV irradiation from 0 h to 24 h. The mean difference in the photocatalytic degradation was found to be significant among the time points (t) at p < 0.05 by two-way ANOVA (t = 7).

In the presence of the undoped and Ag-doped TiO_2_NPs, when the dye solutions were exposed to UV-A light irradiation, a change in MB dye concentration was observed. The peak intensity at 662 nm for the MB dye decreased with light irradiation time. 5 mL of the aliquot was collected and centrifuged after each 1 h interval, and then the supernatant was used to measure the intensity of MB dye. The removal efficiency of the MB dye was found to be 78%, 87%, 91% and 92% for TiO_2_NPs, 1%Ag-TiO_2_NPs, 3%Ag-TiO_2_NPs, and 5%Ag-TiO_2_NPs, respectively, as calculated from the experimental observation of UV-visible absorption spectra. The reaction rates for the photocatalytic decomposition of MB dye were found to fit with the pseudo-first-order reaction kinetics. The reaction rate constant obtained from the ln(*A*_0_/*A*_*t*_) vs.*t* (time) plot, as shown in Figure 10 was 1.03 × 10^−3 ^min^−1^, 1.46 × 10^−3 ^min^−1^, 1.7 × 10^−3 ^min^−1^ and 1.81 × 10^−3 ^min^−1^ for TiO_2_NPs, 1%Ag-TiO_2_NPs, 3%Ag-TiO_2_NPs, and 5%Ag-TiO_2_NPs, respectively. A test of statistical significance using ANOVA indicated the p-value for the sample factor (TiO_2_NPs, 1%Ag-TiO_2_NPs, 3%Ag-TiO_2_NPs, 5%Ag-TiO_2_NPs) was 0.0974. This indicates that the effect of different samples on methylene blue dye degradation is not statistically significant at the 5% level of significance (LOS). However, it is statistically significant at the 10% LOS. In contrast, the p-value for the time factor was 0, which is highly significant at both 5% and 10% LOS. This suggests that time has a statistically significant effect on methylene blue dye degradation. Thus, the choice of NPs significantly impacts the degradation process at the 10% LOS while the duration of the process plays a crucial role in determining the degradation efficiency.

**FIGURE 11:**
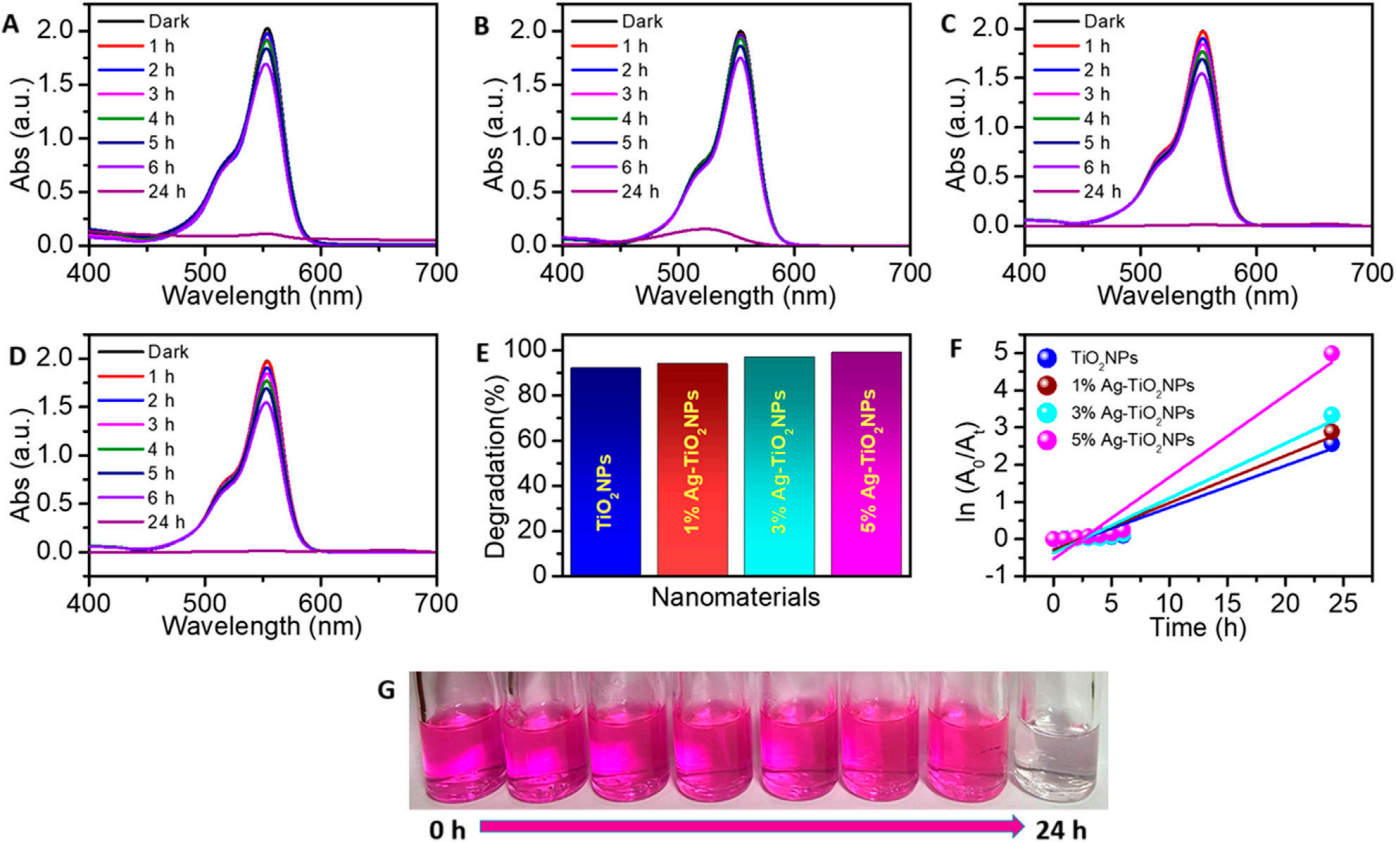
RhB dye degradation under the exposure of UV light by: **(A)** TiO_2_NPs; **(B)** 1%Ag-TiO_2_NPs; **(C)** 3%Ag-TiO_2_NPs; **(D)** 5%Ag-TiO_2_NPs; **(E)** Dye removal efficiency; **(F)** Kinetic study for the photodegradation of the RhB dye; and a **(G)** representative digital picture of the RhB dye degradation under UV irradiation from 0 h to 24 h. The mean difference in the photocatalytic degradation was found to be significant among the time points (t) at p < 0.05 by two-way ANOVA (t = 7).

A similar trend was noted for the degradation of the RhB dye where the removal efficiency was found to be 92%, 94%, 97% and 99% for TiO_2_NPs, 1%Ag-TiO_2_NPs, 3%Ag-TiO_2_NPs, and 5%Ag-TiO_2_NPs, respectively, as calculated from the experimental observation of UV-visible absorption spectra with a maximum absorption wavelength of RhB at 554 nm. The reaction rates for the photocatalytic decomposition of RhB dye were found to fit with the pseudo-first-order reaction kinetics. The reaction rate constants obtained from the ln(*A*_0_/*A*_*t*_) vs.*t* (time) plot, as shown in Figure 11 were 1.88 × 10^−3 ^min^−1^, 2.12 × 10^−3 ^min^−1^, 2.46 × 10^−3 ^min^−1^ and 3.67 × 10^−3 ^min^−1^ for TiO_2_NPs, 1%Ag-TiO_2_NPs, 3%Ag-TiO_2_NPs, and 5%Ag-TiO_2_NPs, respectively. The ANOVA showed that both time factor (p-value is 2.0 × 10^−16^) and sample factor (p-value is 0.0036) have a statistically significant effect on dye degradation at 5% LOS. The extremely high F-value for time indicates a strong time-dependent increase in degradation, while the significant effect of samples suggests variation across different sample types.

Earlier reports suggested that the band gap reduction associated with the red shift due to metal doping increased photocatalytic activity (Asrafuzzaman et al., 2023). The increase in Ag doping percentage increased the photocatalytic efficiency of the TiO_2_NPs significantly. Ali et al. (2023) speculated that the plausible reason for this phenomenon might be the lowering of the band gap energies and the crystallite size.

**FIGURE 12:**
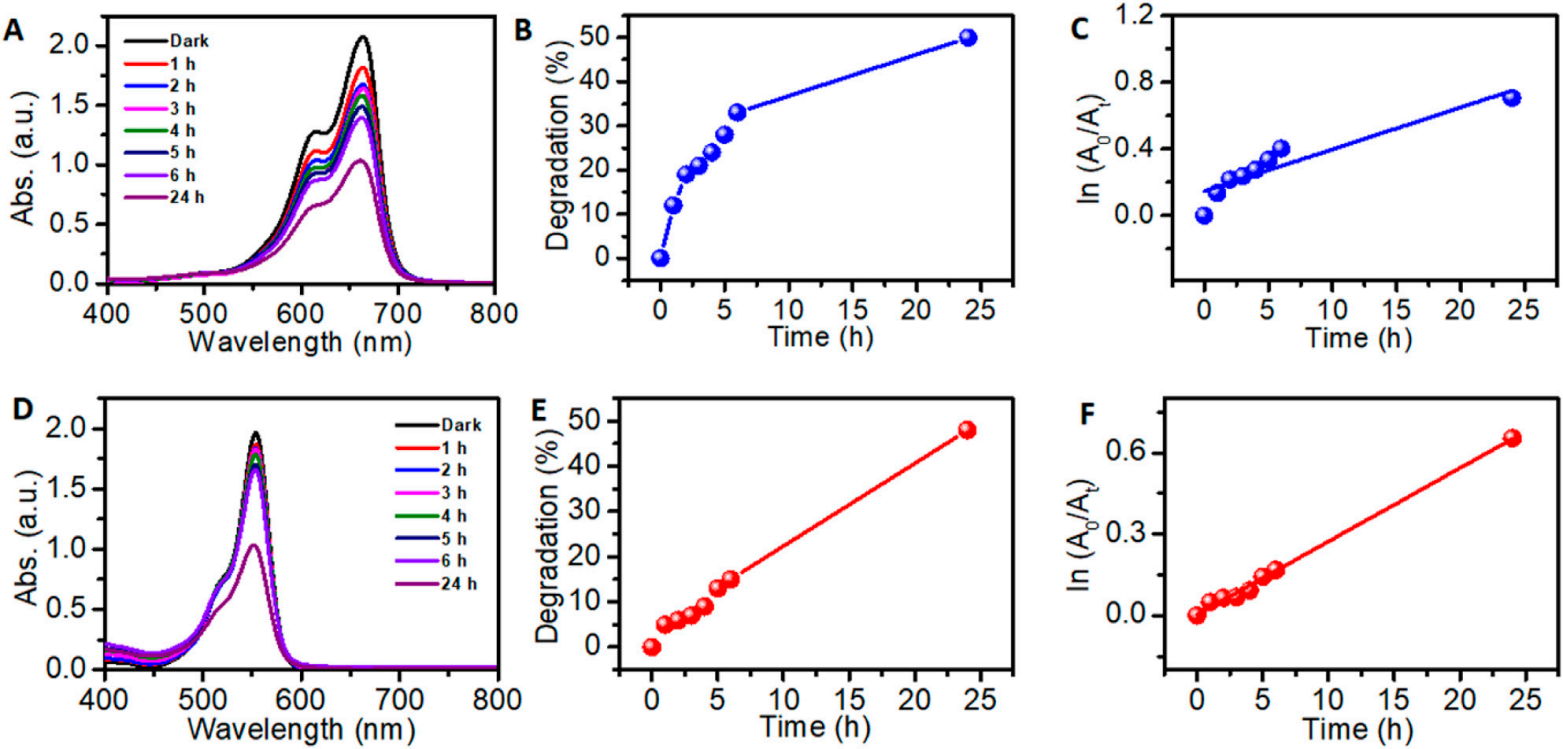
Dye degradation in the presence of 1 mM EDTA-Na_2_ as a scavenger under the exposure of UV light. MB dye degradation by: **(A)** 5%Ag-TiO_2_NPs; **(B)** Dye removal efficiency; and a **(C)** Kinetic study for photodegradation. RhB dye degradation by **(D)** 5%Ag-TiO_2_NPs; **(E)** Dye removal efficiency; and a **(F)** Kinetic study for the photodegradation. The mean difference in the photocatalytic degradation was found to be significant among the time points (t) at p < 0.05 by two-way ANOVA (t = 7).

In order to confirm the mechanism of superior dye degradation by 5%Ag-TiO_2_NPs under UV irradiation, a quenching experiment was carried out by adding 1 mM EDTA-Na_2_ as a scavenger. The photodegradation of MB and RhB dye reduced to 50% and 48% with a rate constant (k_2_) equivalent to 4.18 × 10^−4^ and 4.5 × 10^−4^, respectively, after 24 h as seen in Figure 12. The ANOVA indicated that both time factor (p-value is 0.0004) and dyes (p-value is 0.0029) have a statistically significant effect at 5% LOS on dye degradation in the presence of EDTA-2Na. The high F-values suggest that degradation varies significantly over time and between the two dyes (MB and RhB). This clearly indicates the role of the photo-generated holes in the VB of TiO_2_NPs in the dye degradation process. The holes can produce •OH from water molecules or OH− ions and may also react directly with the dye molecules, leading to its degradation. Hence, EDTA-Na_2_ mediated scavenging of holes suppressed the generation of ROS and/or direct oxidative degradation of MB and RhB dyes (Verma et al., 2019).

**FIGURE 13:**
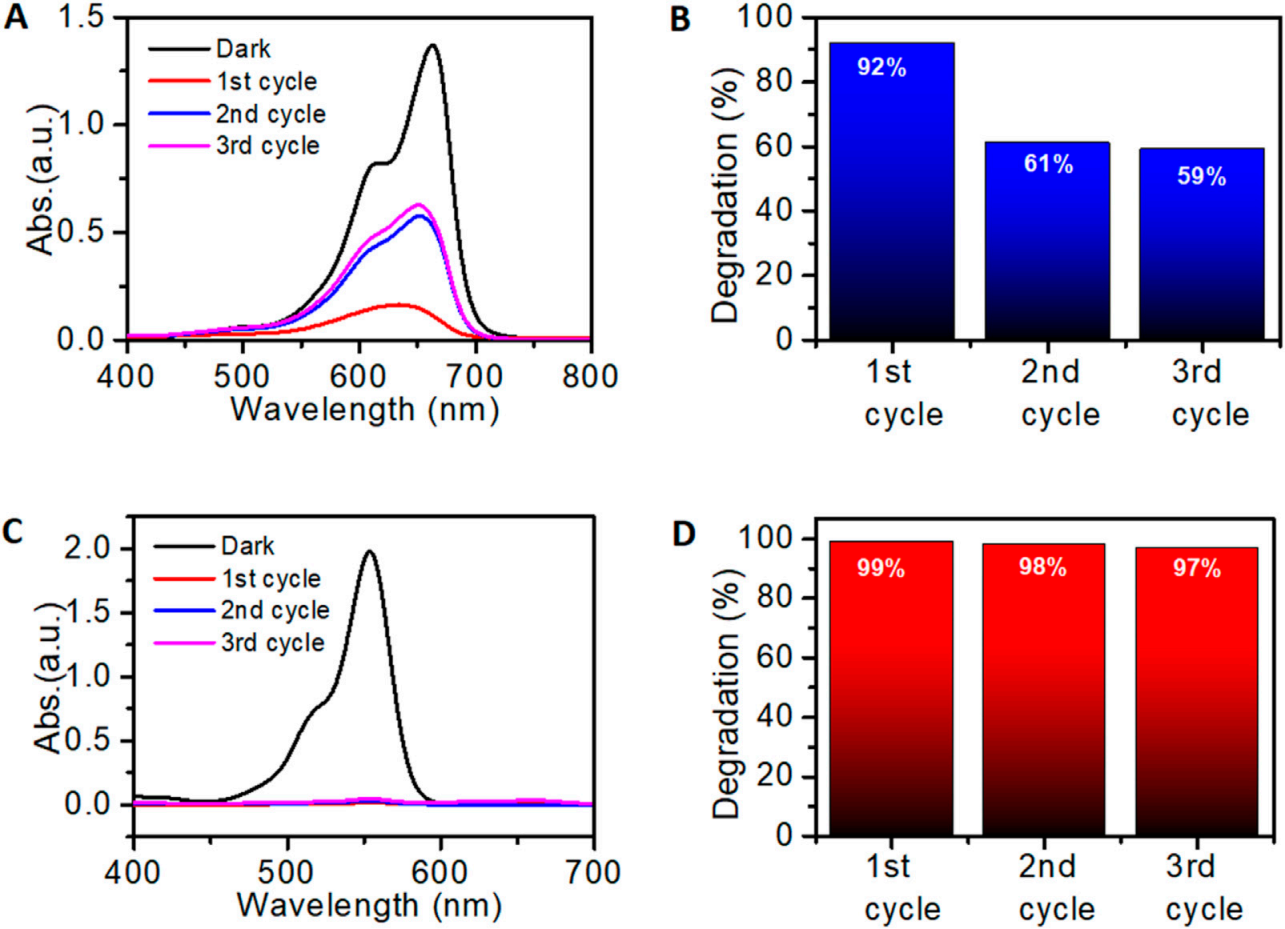
Reusability of 5%Ag-TiO_2_NPs for the degradation of dyes. **(A)** MB dye degradation in three cyclic runs; **(B)** MB dye removal efficiency; **(C)** RhB dye degradation in three cyclic runs; and **(D)** RhB dye removal efficiency. The mean difference in the photocatalytic degradation was not significant among the cycles **(C)** at p > 0.05 by two-way ANOVA (c = 3).

**FIGURE 14:**
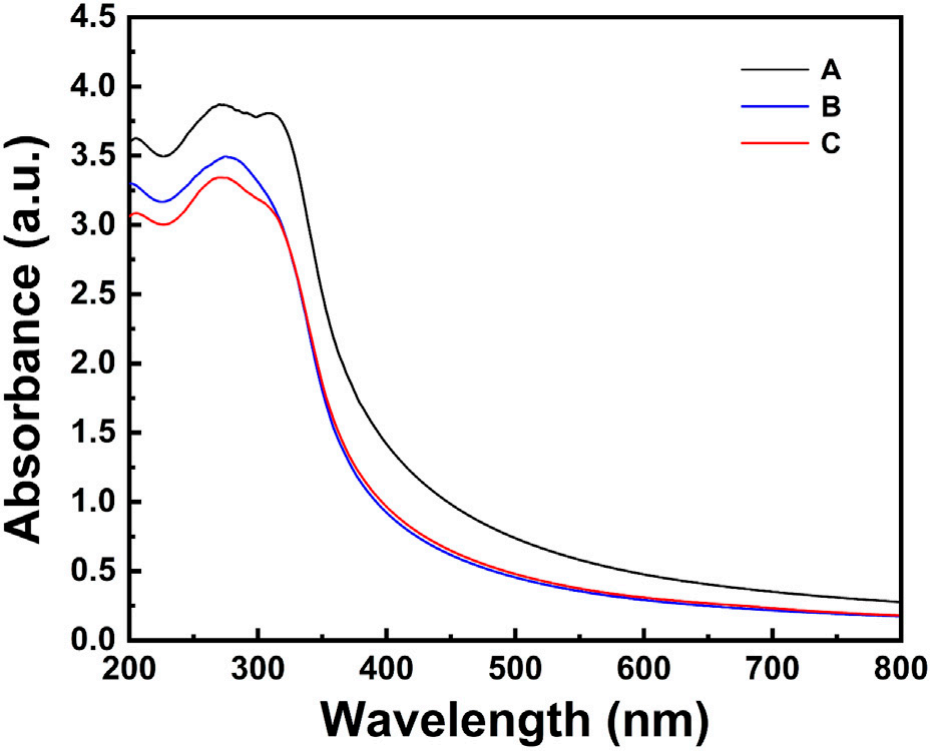
The UV–vis absorbance spectra of 5%Ag-TiO_2_NPs. (A) Before and after photocatalytic degradation of (B) MB and (C) RhB dyes after 3 cyclic runs.

Reusability of photocatalysts is a significant parameter that can result in the reduction of the overall cost of the treatment process. The recyclability is shown in Figure 13. The 5%Ag-TiO_2_NPs were investigated for photodegradation of MB and RhB dyes through three successive runs keeping the conditions the same in each cycle. After each cycle that was for 24 h, the degradation efficiency of the catalyst was evaluated. MB dye degradation was 92% in the first cycle which reduced to 61% and 59% in the second and third cycles, respectively. However, the RhB dye degradation was fairly stable in all three cycles which was equivalent to 99%, 98%, and 97% in the first, second and third cycles, respectively. These results rationalize that the 5%Ag-TiO_2_NPs remained effective for the first cycle, while it was reduced in the successive cycles that might be attributed to slight inactivation caused by surface adsorption of small fragmented species during the degradation process. The ANOVA indicated that neither cycles nor the dyes had a statistically significant effect on the degradation efficiency. The high F-value for the dyes suggests more variability between the dyes, but it is not significant at the 5% LOS. The 5%Ag-TiO_2_NPs was examined for its stability by UV–vis spectroscopy. Figure 14 shows the UV-vis absorbance spectra of the 5%Ag-TiO_2_NPs before reaction which was very similar to its spectra after three cycles of photocatalytic degradation reactions for MB and RhB dyes. This indicates the strong stability of the photocatalyst. A similar observation was reported during the degradation of MB and RhB dyes by ZnS/Ag/CoFe_2_O_4_ nanocomposites (Palanisamy et al., 2020).

**FIGURE 15:**
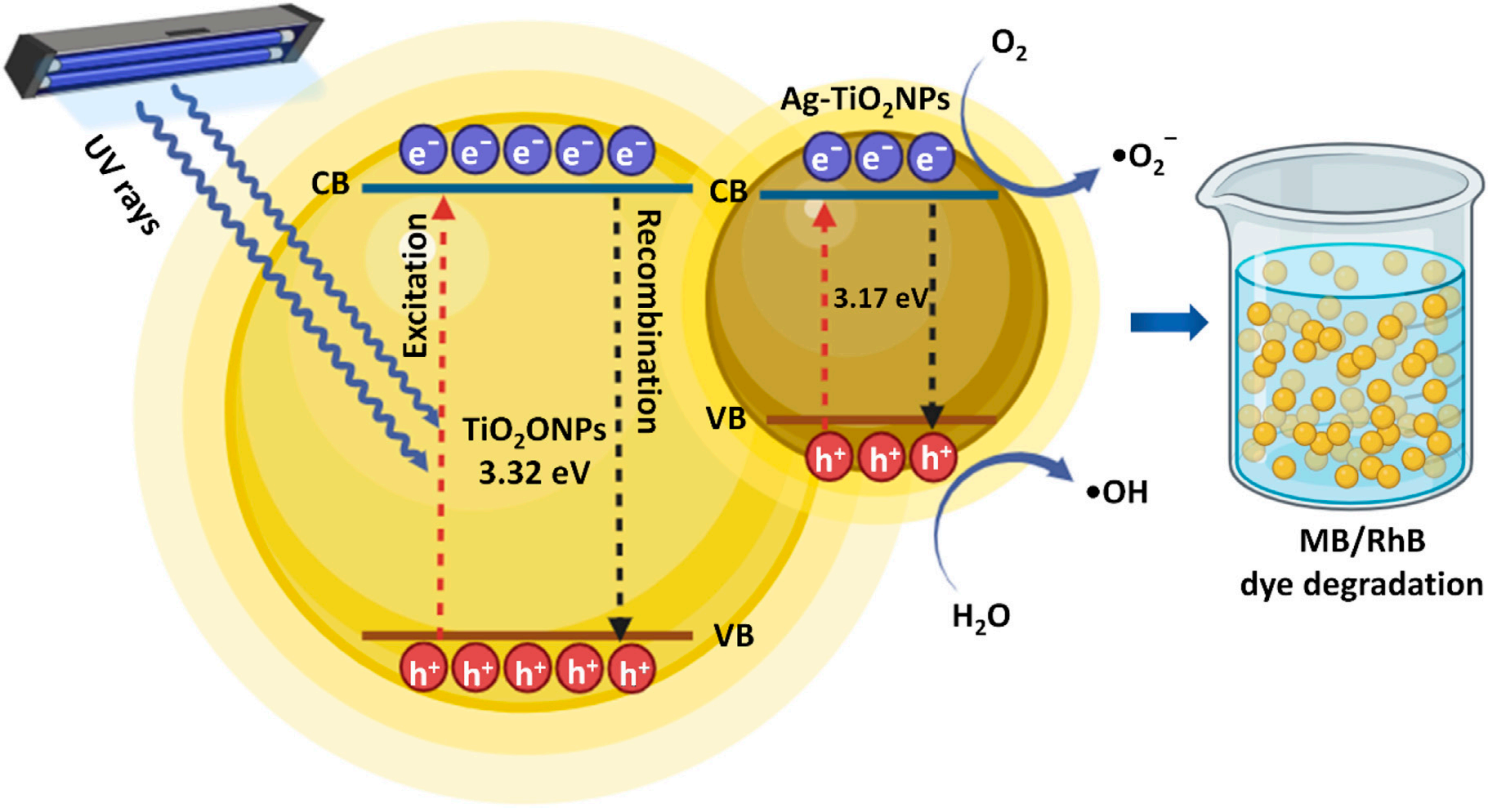
Schematic representation of the mechanism behind the photocatalytic MB dye degradation using undoped and Ag-doped TiO_2_NPs.

Figure 15 schematically represents the mechanism behind the increased photocatalysis by the Ag-doped TiO_2_NPs by considering the existing literature (Rathi et al., 2023). It is important to understand the role of Ag-doping in the enhancement of photocatalytic activity of the TiO_2_NPs. Ag is a stable dopant with maximum electrical as well as thermal conductivity. Hence, it is a preferred electron moderator in the Z-scheme photocatalyst structure that can also serve as a photosensitizer facilitating the generation of stable electron-hole pairs using low-energy photons (Kanakaraju et al., 2022). Ag doping in TiO_2_NPs reduces the recombination of the electron-hole pairs and extends the light absorption range of a TiO_2_ photocatalyst towards visible light. There are two distinct ways by which the Ag enhances photocatalytic activities. Firstly, Ag acts as an electron trap and captures the electrons that are transferred from the conduction band of the TiO_2_ semiconductor. These electrons are then transferred to the oxygen resulting in the generation of the superoxide radicals (•O_2_^‾^). The photogenerated holes in the valence band (VB) associated with the TiO_2_ react with water molecules generating hydroxyl radicals (•OH) that photocatalytically oxidizes the hazardous dyes. Secondly, an Ag dopant induces an SPR effect that extends the light absorption to the visible light region and improves the photocatalytic efficiency of TiO_2_ (Chakhtouna et al., 2021).

It is speculated that Ag doping improves the photocatalytic potential of TiO_2_ in multiple ways. It modifies the morphology of the TiO_2_ resulting in its higher affinity for dissolved oxygen. Ag reduces the recombination of charge carriers. Also, the plasmonic effect-electron injection is a direct result of Ag doping. These features cumulatively result in higher reactive oxygen species (ROS) generation from the photo-excited electrons of TiO_2_ and adsorbed O_2_. Ag-doped TiO_2_NPs, with an improved affinity for dissolved O_2_ and increased surface area, resulted in enhanced O_2_ adsorption due to more attraction of the dissolved O_2_ from the solution towards its surface (Cruz et al., 2022).

In another study, microclusters of TiO_2_NPs were generated employing a simultaneous electrospinning and electrospraying strategy which were further incorporated into a nanofibrous network. A 98% MB removal efficiency was speculated to be attributed to the enhanced specific surface area that resulted in higher hydroxyl radical (•OH) generation which is a strong oxidizing agent. This eventually degraded the adsorbed MB molecules by enhancing surface interactions (Pahasup-anan et al., 2018). This confirms the involvement of the interlocking components on the TiO_2_NPs surface in enhancement of the photocatalytic degradation of the adsorbed dyes. Likewise, glutathione (GHS) capping enhanced the photocatalytic dye degradation efficiency of ZnS, CdS and inverted type-I ZnS@CdS core-shell quantum dots (QDs). Superior quantum confinement of both the hole and electron in three dimensions resulted in an increase in the effective band gap of the nanomaterials with reducing crystallite size. Researchers speculated that the enhanced photocatalytic activity might be attributed to the decreased electron-hole recombination rate by separation of charge through the formation of the heterojunction (Bhuvaneswari et al., 2020). Thus, the above observations strongly rationalize the role of catechin capping in enhancing the photocatalytic performance, stability, charge separation, and surface interactions clearly evident from the comparative Table 3.

**TABLE 3:** Comparison of characteristics and dye degradation efficiency for various TiO_2_-based photocatalysts.

Photocatalysts	Size (nm)	Shape	Bandgap (eV)	Dye	Degradation efficiency (%)	Reference
TiO_2_NPs	23.8	Spherical	3.32	MB	40.10	Komaraiah et al., 2020
Degussa P25 TiO_2_NPs	38.5	Irregular	—	MB	>98	Pahasup-anan et al., 2018
Ag/TiO_2_NPs	20–30	Spherical	2.47	MB	94	Rathi et al., 2023
TiO_2_NPs, Ag-TiO_2_NPs	18	Cuboid, spherical	3.22, 2.67	MB	66, 97	Abbad et al., 2020
Ag-Zn doped TiO_2_NC	5.66	Variable	3	MB, MO	90, 93	Ali et al., 2023
TiO_2_-P25 Degussa, Ag/TiO_2_	100-1,000	Granular	3.2, 1.7	MB	97.6, 60.6	Cruz et al., 2022
PDA/PEI@TiO_2_@P-HSM composites	—	—	2.1	RhB	90	Chen et al., 2024
TiO_2_, Ag/MoO_3_/TiO_2_	11.52, 4.25	—	3.24, 2.89	MO	82.43	Kader et al., 2022
TiO_2_NPs, Ag-TiO_2_NPs	15.55, 14-16	Irregular	3.32, 3.17-3.20	MB, RhB	78–92, 92–99	Current research

Note: MO, methyl orange; PDA, Polymerized dopamine (DA); PEI, polyethyleneimine.

## 4 Conclusion

Ag doping in TiO_2_NPs resulted in the alteration of the energy band gap and zeta potential. The nanoparticles were irregular in shape having a predominant TiO_2_ anatase phase. This is the first report for the synthesis of catechin-mediated Ag-doped TiO_2_NPs that showed promising photocatalytic MB and RhB dye degradation under UV radiation. The reaction rate constant increased from 1.03 × 10^−3 ^min^−1^ to 1.81 × 10^−3 ^min^−1^ for MB and 1.88 × 10^−3 ^min^−1^ to 3.67 × 10^−3 ^min^−1^ for RhB with an increase in the concentration of the Ag dopant in the TiO_2_NPs. The generation of •O_2_^‾^ and •OH radicals was speculated to play a significant role in the degradation of the MB and RhB molecules. Hence, catechin-synthesized undoped and Ag-doped TiO_2_NPs can be considered promising nanomaterials for treating toxic dye-contaminated wastewater.

Highly efficient water treatment technologies can be developed in the future by ensuring its reusability which will make the process more cost effective. Future studies involving recyclability and reusability with facilitate the expansion of the use of nanotechnology in the area of wastewater treatment. Moreover, “real-time monitoring models” should be developed to assess the efficiency of the nanomaterials and the degradation pathways of the specific pollutants. A real-time method will help to predict whether the end-products are toxic or safe. Gaining better knowledge regarding the intermediates and their properties will help to evaluate the importance of nanotechnology for practical wastewater treatment applications.

## Data Availability

The original contributions presented in the study are included in the article, further inquiries can be directed to the corresponding author.
